# Abnormal EEG Effects of Acute Apomorphine Injection in 5xFAD Transgenic Mice Are Partially Normalized in Those Chronically Pretreated with Apomorphine: The Time–Frequency Clustering of EEG Spectra

**DOI:** 10.3390/biomedicines12112433

**Published:** 2024-10-23

**Authors:** Vasily Vorobyov, Alexander Deev

**Affiliations:** 1 Institute of Cell Biophysics, Russian Academy of Sciences, Pushchino 142290, Moscow Region, Russia; 2 Institute of Theoretical and Experimental Biophysics, Russian Academy of Sciences, Pushchino 142290, Moscow Region, Russia; aadeev@gmail.com

**Keywords:** electroencephalogram, cortex, hippocampus, ventral tegmental area, substantia nigra

## Abstract

Background: In experimental and clinical studies of pharmacological treatments for Alzheimer’s disease (AD), the electroencephalogram (EEG) frequency spectrum approach has demonstrated its efficacy in determining the characteristics of pathological changes in the functioning of different cerebral structures, interconnections between them, and disturbances in the brain neurotransmitter systems. The main results have been obtained in frames of traditionally used so-called “classical” EEG frequency bands: delta, theta, alpha, and beta. Objective: This unified approach simplifies comparing data from different studies but loses the dynamic peculiarities of the effects because of their time-dependent transition through the borders of the “classical” bands. Methods: In this study on non-narcotized freely moving 5xFAD transgenic mice, a model of AD, chronically pretreated with a non-selective dopamine (DA) receptor agonist, apomorphine (APO), we analyze the transitory EEG effects of acute APO injection in different brain areas by use of our “time–frequency” clustering program. The acute injection of APO was used to compare DA receptor sensitivity in 5xFAD mice pretreated with either APO or saline vs. wild-type (WT) mice pretreated with saline. Results: After acute APO injection, the clusters of enhanced EEG activity centered in the theta–alpha frequency range observed in WT mice disappeared in 5xFAD mice pretreated with saline and practically recovered in 5xFAD mice pretreated with APO. Conclusions: In 5xFAD mice pretreated with saline, the sensitivity of DA receptors was disturbed; chronic APO pretreatment mainly recovered this characteristic in 5xFAD mice. The “clustering” of pharmacological EEG effects and their time-dependent transition between classical frequency bands is a new effective approach for analyzing cerebral neurotransmission in neurodegenerative pathologies.

## 1. Introduction

Alzheimer’s disease (AD) is characterized by memory impairment associated with neurodegenerative processes in the brain as well as pathologically enhanced levels of amyloid plaques and neurofibrillary tangles [1]. In AD, an evident disruption of neuronal connectivity [2] and synaptic transmission [3] and the loss of neurons [4] and synapses [5] have been revealed. Disturbances in neuronal synaptic connectivity are considered “a novel target for therapeutic intervention” in AD [6], predominantly associated with the dopaminergic system losing its neurons in neurodegenerative diseases, particularly AD [7,8]. One well-known pharmacological approach is associated with the use of dopamine (DA)-mimetic apomorphine (APO) as a drug that has been shown to promote amyloid-β degradation after chronic subcutaneous injections in transgenic mice, a model of AD [9]. In addition, the beneficial effect of APO in AD is expected to be supported by other mechanisms involved in amyloid-β genesis and its destruction [10,11], particularly associated with APO’s mitigation of intracellular stress responses inflicted by hyperphosphorylated tau [12]. Thus, after chronically administrated APO, neuronal interactions in the brain may normalize, which is partially confirmed by the rescue of damaged striatal dopaminergic terminals by APO and its dose-dependent prevention of MPTP toxicity [13]. The evident potential of chronically repeated dopaminergic stimulation allowed for the development of a new approach based on continuous dopaminergic therapy for neurodegenerative diseases [14].

In AD, imbalances in intra-/inter-neuronal circuits of different brain structures [15] (see [16] for review) have been shown to result in the modification of oscillations in affected networks [17,18]. Synaptic transmembrane currents of neuronal populations producing oscillations of extracellular fields are considered a source of the electroencephalogram (EEG) [19]. Changes in EEG frequency compositions have been shown to correlate with the evolution of AD pathology [20], having the potential to reveal its early symptoms and optimal treatment [21,22]. So far, EEG data have been analyzed predominantly in frames of traditionally used so-called “classical” EEG frequency bands: delta, theta, alpha, and beta [23]. This unified approach simplifies comparing data from different studies but loses the dynamic peculiarities of the effects because of their time-dependent transition through the borders of the “classical” frequency bands. The latter is mainly expected in studies of compounds affecting DA receptors and other neurotransmitters in various brain areas [24], leading to additional difficulties in the evaluation of pharmacological effects on neuronal nets and their function.

In this study on non-narcotized freely moving 5xFAD transgenic mice, a model of AD, chronically pretreated with APO, we analyze the transitory EEG effects of acute APO in the cortex, hippocampus, and dopamine-containing areas (ventral tegmental area and substantia nigra) by use of our “time–frequency” clustering program. The acute injection of APO was used to compare DA receptor sensitivity in 5xFAD mice pretreated with APO or saline and in wild-type (WT) mice pretreated with saline.

## 2. Materials and Methods

### 2.1. Experimental Animals

Seventeen seven-month-old male 5xFAD transgenic mice (body weight: 30.4 ± 3.2 g) with five familial AD mutations maintained with a C57BL/6J genetic background and eight wild-type (WT) littermates (body weight: 37.2 ± 4.8 g) were used in this study. All mice were originally obtained from the Center for Collective Use of the Institute of Physiologically Active Compounds RAS (Chernogolovka, Russian Federation) and were housed in a standard environment (12 h light/dark cycle, 22–25 °C RT, 50–55% relative humidity) with food and water ad libitum. Up to the age of six months, they were kept in groups of five animals and then individually for one month. Afterwards, the 5xFAD mice were randomly divided into two cohorts, receiving one-month daily subcutaneous (s.c.) treatment with either saline (n = 9) or APO (Sigma, Milan, Italy) at a dose of 1 mg/kg (n = 8), while in the WT group (n = 8), only saline was chronically injected. The procedures were carried out in accordance with the “Guidelines for accommodation and care of animals. Species-specific provisions for laboratory rodents and rabbits” (GOST 33216–2014), in compliance with the principles enunciated in Directive 2010/63/EU on the protection of animals used for scientific purposes and approved by the local Institute Ethics Review Committee (protocol No. 52, 18 September 2020). All efforts were made to minimize the number of animals and their suffering. 

### 2.2. Electrode Implantation and Recording of EEG

After one month of daily injections of either APO or saline, each mouse was anesthetized with a subcutaneous (s.c.) injection of a combined solution of tiletamine/zolazepam (25 mg/kg, Zoletil^®^, Virbac, Carros, France) and xylazine (2.5 mg/kg, Rometar^®^, Bioveta, Ivanovice na Hané, Czech Republic). After control testing of the sufficient depth of narcosis (completely relaxed posture, no responses to ear/tail pinching or blowing on the eyes), the animal was placed in a computerized 3D stereotaxic StereoDrive (Neurostar, Tubingen, Germany) on a heating pad supporting the body temperature at the optimal level for the whole period (around 3 h) of the surgery. Before implantation of the electrodes, the upper and both sides of the head were shaved, and the skin and all muscles on the upper lateral surface of the skull were removed. All bleeding points were blocked, and the skull surface was cleaned and dried. At the next step of the surgery, the skull surface was marked, and drilled holes of 0.4 mm in diameter were prepared according to the chosen coordinates.

Recording electrodes were placed in the left frontal cortex (MC) and hippocampus (HPC) with coordinates as follows: AP +1.1 mm anterior to bregma, ML 1.5 mm lateral to midline, and DV −0.75 and −2.75 mm depths from skull surface; in the left ventral tegmental area (VTA), AP −3.1, ML −0.4, and DV −4.5; and in the right substantia nigra (SN), AP −3.2, ML +1.3, and DV −4.3, according to the stereotaxic atlas of the mouse brain [25]. 

The electrodes for EEG recordings from VTA and SN were constructed from two glued (3M Vetbond TM Tissue Adhesive, MN, USA) insulated nichrome wires 100 µm in diameter (NK, Nanjing, China), bared both at the tips of 100 µm. Two wires in combined electrodes used for EEG recordings from MC and HPC were different in length of 200 µm according to the depth of these brain areas’ positions (see their coordinates above). The reference and ground electrodes (stainless steel wires, 0.4 mm in diameter) were inserted into the skull symmetrically above the cavities between the cerebellum and cerebrum (AP: −5.3, ML: ±1.8, DV: −0.5). All electrodes were positioned by using the computerized 3D stereotaxic StereoDrive. Afterwards, they were fixed to the skull with dental cement and soldered to the corresponding pins of a mini-connector (Sullins Connector Solutions, San Marcos, CA, USA). For post mortem verification of the electrode tip location through coagulation of the adjacent tissue, an anodal current of 10 µA, 1 s was used. Details of other manipulations with brain tissue are described in [26].

After electrode implantation, each animal was continued to be kept in an individual cage. Three days later, all mice were adapted for four days (1 h/day) to both an experimental cage located in an electrically shielded chamber and to a cable plugged into a digital amplifier (Neurosoft Ltd, Ivanovo, Russia). Usually, on day 8 after the surgery, a baseline EEG was recorded for 30 min, starting 20–30 min after placing the animal into the box. EEG recordings were continued for 120 min after s.c. injection of either saline (control) or freshly dissolved APO on the next day at a dose of 1.0 mg/kg. All experiments were performed predominantly from 9 am to 6 pm in daylight, with an additional artificial light source keeping illumination at a stable level.

### 2.3. Computation of EEG Frequency Spectrum and Its Clustering 

EEG signals measured between the active and reference electrodes were amplified, filtered (0.1–35 Hz), and sampled (1 kHz) on-line. EEG fragments containing artefacts and epileptic spikes were automatically and manually rejected by the custom-developed software. The frequency spectra of successive 12 s EEG epochs were analyzed using a modified version of the period–amplitude analysis [27], which, contrary to the Fourier transform, was not affected by the well-known non-stationary nature of the EEG signals. The values in each frequency band were averaged in every successive 10-min interval for further “time–frequency” clustering analysis. In this study, twenty-five sub-bands in a range of 0.48–31.5 Hz were analyzed: 0.48–0.53 (0.5), 0.83–0.92 (0.9), 1.20–1.33 (1.3), 1.59–1.76 (1.7), 1.99–2.20 (2.1), 2.42–2.67 (2.5), 2.86–3.17 (3.0), 3.34–3.69 (3.5), 3.83–4.24 (4.0), 4.36–4.82 (4.6), 4.92–5.44 (5.2), 5.52–6.10 (5.8), 6.17–6.82 (6.5), 6.87–7.59 (7.2), 7.62–8.43 (8.0), 8.45–9.34 (8.9), 9.37–10.36 (9.9), 10.40–11.49 (10.9), 11.56–12.77 (12.2), 12.90–14.26 (13.6), 14.49–16.01 (15.3), 16.43–18.16 (17.3), 18.93–20.93 (19.9), 22.47–24.83 (23.6), 28.50–31.50 (30.0). The sub-bands are marked in figures by their center (mean) frequency values (see in brackets above). 

The relative differences in the averaged EEG spectra in each mouse separately for the WT and 5xFAD groups, obtained in the experiments with saline (day 1) and APO (day 2) in each group, were estimated as (APO − saline)/saline, in percentages, providing the evaluation of APO effects. To analyze and compare the effects in the time–frequency matrix, the image processing algorithms were applied. For this purpose, the distributions of the calculated values were resized by bilinear interpolation and smoothed out using a two-dimensional Fourier filtration followed by the selection of areas (“clusters”) separately for positive and negative values. Finally, equipotential lines built from significant APO vs. saline differences in each sub-band allowed the highlighting of appropriate clusters and the estimating of their size and position. 

### 2.4. Statistics

The significance (*p* < 0.05) of the APO vs. saline differences in the EEG spectra averaged for 10 min in frames of the analyzed frequency subranges was estimated by the Mann–Whitney non-parametric U-test. All data are shown as mean ± SE.

## 3. Results

### 3.1. Baseline EEG

**Figure 1:**
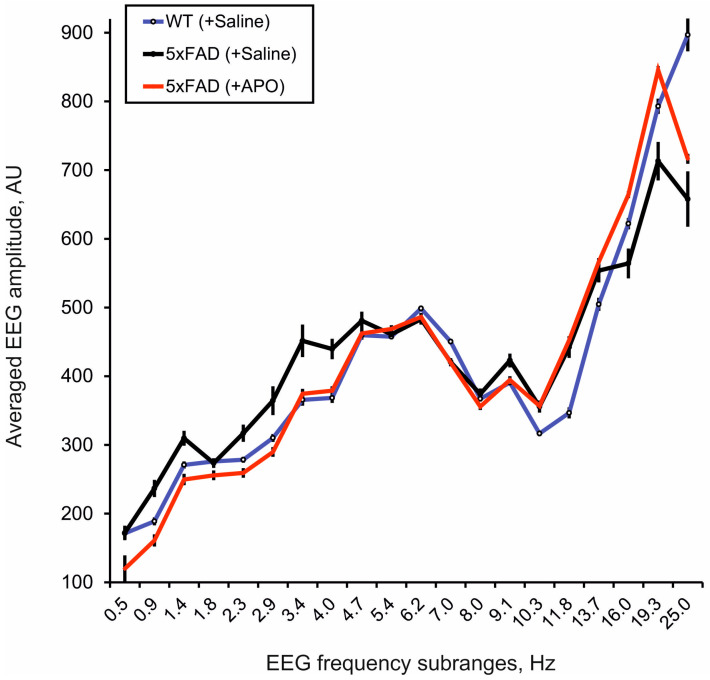
Frequency spectra of EEG recorded from the motor cortex in 30 min baseline intervals in seven-month-old 5xFAD mice chronically pretreated with either saline (black line, n = 9) or APO (red line, n = 8) and in WT littermates (blue line, n = 8) pretreated with saline. Ordinate is an averaged EEG amplitude in the individual frequency subrange normalized to the sum of the amplitudes in the whole spectra, in arbitrary units (AU); abscissa indicates different frequency subranges indicated by their central frequencies, in hertz (Hz); vertical bars are ±SEM.

**Figure 2:**
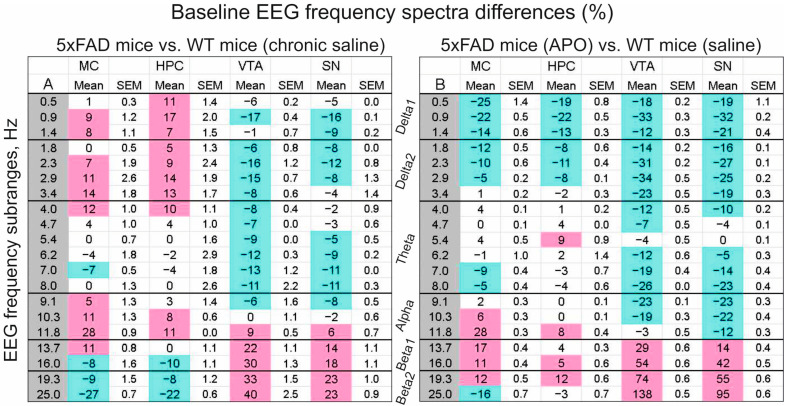
Relative differences (in %) between frequency spectra of baseline EEG recorded for 30 minutes from different brain structures in 5xFAD mice chronically pretreated with either saline ((**A**), n = 9) or APO ((**B**), n = 8) vs. WT littermates (n = 8) pretreated with saline. Notes: (1) The values characterize 30 min baseline intervals as a ratio of averaged EEG signal per frequency sub-band for 5xFAD and WT mice calculated as (5xFAD − WT)/WT, in %; (2) colored cells are significantly (*p* < 0.05, U-test) elevated (pink) and lowered (blue) levels; (3) MC, HPC, VTA, and SN denote the cortex, hippocampus, ventral tegmental area, and substantia nigra, respectively.

The EEG frequency spectra averaged in a 30 min baseline period were different in the MC of 5xFAD vs. WT mice chronically pretreated with saline (see Figure 1, black vs. blue lines, respectively, and MC in Figure 2A), while these differences were modified by chronic pretreatment with APO (see Figure 1, red line, and MC in Figure 2B). 

The 5xFAD vs. WT mice were characterized by significantly enhanced delta waves in the cortex and hippocampus (Figure 2A, MC and HPC, respectively) with concomitant attenuation of beta 2 oscillations. In contrast to that, in the VTA and SN, delta–theta activity was suppressed, which was accompanied by significant amplification of the beta. 

In contrast, delta–theta activity suppression and beta amplification were observed in the VTA and SN. Chronic APO pretreatment in 5xFAD mice eliminated the delta power enhancement revealed in baseline EEG from MC and HPC in the mice pretreated with saline and even significantly suppressed the slow “pathological” activity (Figure 2B). Chronic pretreatment with APO amplified the differences in the frequency spectra of baseline EEG from the VTA and SN observed in 5xFAD vs. WT mice pretreated with saline (see Figure 2A).

### 3.2. Clustering of Acute APO Effects in EEG Frequency Spectra 

**Figure 3:**
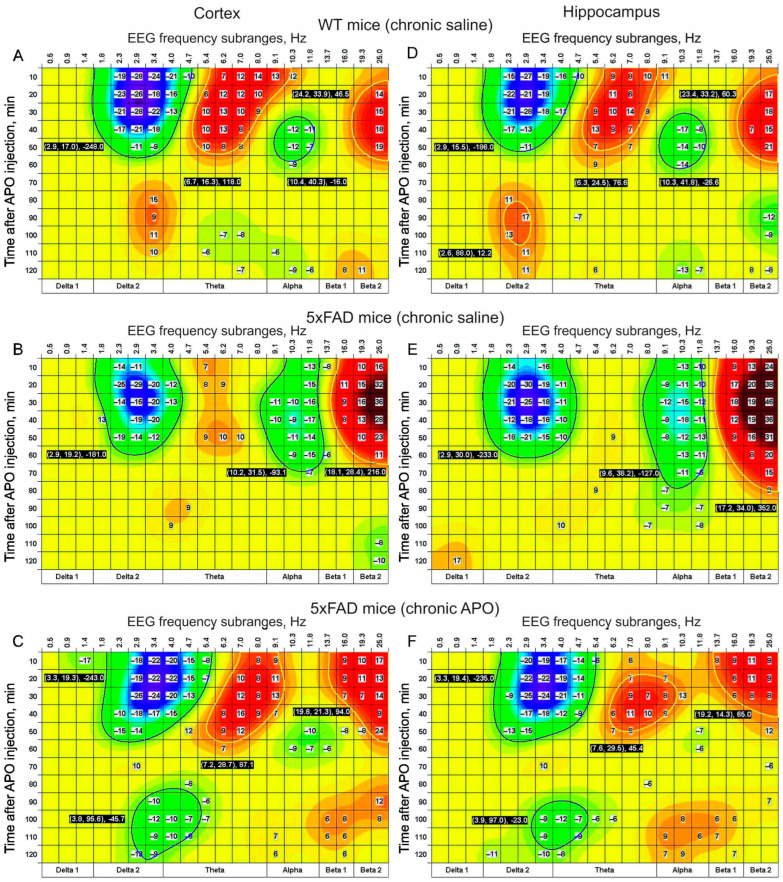
Time–frequency clustering of EEG effects of acute APO (vs. saline) in the cortex (**A**–**C**) and hippocampus (**D**–**F**) in WT and 5xFAD mice (n = 8 and 9, respectively) chronically pretreated with saline ((**A**,**D**) and (**B**,**E**), respectively) and 5xFAD mice (n = 8) chronically pretreated with APO (**C**,**F**). The clusters are colored in accordance with the scale positioned on the right and denote roughly the elevation and lowering (brighter and darker colors, respectively) of EEG activity. White numbers contrasted by a black base denote the pick centers of the clusters (frequency and time) and their integrated size/power. The differences in statistical parameters (mean and SEM) are in Table A1 and Table A2.

In WT mice chronically pretreated with saline, acute APO s.c. injection mainly produced early (up to 40–50 min) significant suppression of delta activity and amplification of the theta in the EEG from the cortex and hippocampus (Figure 3A,D). In 5xFAD mice pretreated with saline, the acute APO effect in the theta band disappeared, while the alpha suppressive tendency observed in WT mice was replaced by a significant and prolonged effect in this band (Figure 3B,E). In 5xFAD mice pretreated with APO, the EEG effects of acute APO were similar to those in WT mice with their expanding theta and alpha bands (Figure 3C,F).

**Figure 4:**
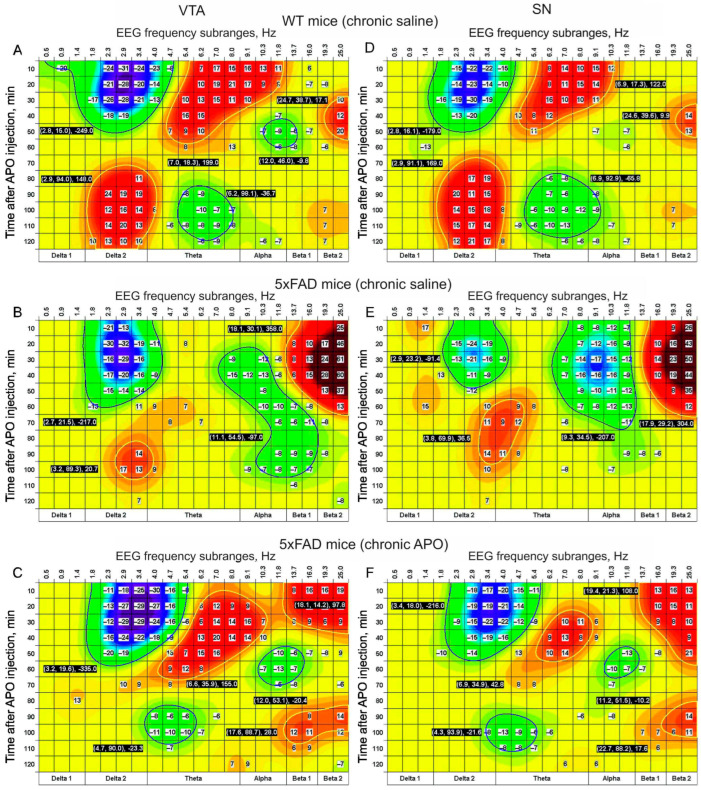
Time–frequency clustering of EEG effects of acute APO (vs. saline) in VTA (**A**–**C**) and SN (**D**–**F**) in WT and 5xFAD mice (n = 8 and 9, respectively) chronically pretreated with saline ((**A**,**D**) and (**B**,**E**), respectively) and 5xFAD mice (n = 8) chronically pretreated with APO (C,F). The clusters are colored in accordance with the scale positioned on the right and denote roughly the elevation and lowering (brighter and darker colors, respectively) of EEG activity. The inserted white numbers contrasted by a black base denote the pick centers of the clusters (frequency and time values) and their integrated size/power. The differences in statistical parameters (mean and SEM) are shown in Table A3 and Table A4.

In the EEG from the VTA and SN, in WT mice chronically pretreated with saline, acute APO produced more powerfully expressed changes in the EEG frequency spectra (A and D, respectively, in Figure 4) than those observed in the MC and HPC (c.f., A and D in Figure 3).

Indeed, the early effect of theta activity amplification expanded to the alpha band and, finally, a cluster of theta–alpha suppression with the center around 95 minutes after acute APO injection (see corresponding values on A and D in Figure 4) was revealed. In 5xFAD mice pretreated with saline, after acute APO injection, the late delta and early theta effects disappeared, while alpha early suppression was observed (Figure 4B,E). In 5xFAD mice pretreated with APO, the EEG effects of acute APO in the VTA were similar to those in WT mice, with the exception of late amplification of the delta (c.f., C and A in Figure 4). In the SN, these 5xFAD mice demonstrated lower levels of changes in the EEG frequency spectra after acute APO injection than those in WT mice (c.f. F and D in Figure 3). These 5xFAD mice demonstrated lower levels of changes in the frequency spectra of the EEG recorded from the SN after acute APO injection compared with those in WT mice (c.f. F and D in Figure 3).

## 4. Discussion

The baseline EEGs recorded from the motor cortex and hippocampus of non-narcotized freely moving 5xFAD mice chronically pretreated with saline were characterized by the significant enhancement in delta activity and attenuation of the beta (see Figure 2). These differences were eliminated (or even inversed) in 5xFAD mice chronically pretreated with APO. In contrast, the differences between 5xFAD mice and WT littermates in the EEG recorded from the DA-containing/producing brain areas, which were characterized by suppressed delta–alpha activity and enhanced beta, were potentiated by chronic APO pretreatment in 5xFAD mice. This differentiation in the influence of chronic APO on different brain regions seems to be indicative of differences in DA-dependent mechanisms involved in the observed EEG changes. Interestingly, in the cortical and hippocampal EEGs in 5xFAD mice chronically pretreated with either saline (Figure 2A) or APO (Figure 2B) vs. WT littermates pretreated with saline, practically no significantly expressed differences in theta activity were observed, whereas in the VTA and SN, evident theta suppression was characteristic of 5xFAD mice. In a first approximation, this is explainable given that the forebrain projections from the DA-containing areas are incorporated into the main pathways of the theta generation [28], and different extents of their activity modification can either attenuate it [29] (e.g., after VTA/SN partial destruction, typical for 5xFAD mice) or even enhance it [30]. 

In 5xFAD transgenic mice chronically pretreated with saline, acute s.c. injection of APO (vs. saline) did not form clusters of time- and frequency-dependent rising of EEG activity centered in the theta band in any of the brain areas studied. This was observed in WT and 5xFAD mice chronically pretreated with saline and APO, respectively (see plates A and D in Figure 3 and Figure 4). The early enhancement of EEG activity in the theta–alpha range after acute APO injection in WT mice coincides with that observed after injection of APO at a comparable dose (0.5 mg/kg) in rats [31]. Interestingly, the late (with 60–70 min latency) rise in EEG delta activity, more powerfully expressed in the DA-containing areas (see plates A and D in Figure 3 and Figure 4), is reminiscent of rats treated with APO at low doses (0.02 and 0.05 mg/kg) [31]. Together, these findings allow the suggestion that the EEG effect of APO is formed by the activation of post- and pre-synaptic DA receptors [32] depending on the pharmacokinetic distribution of APO in different brain areas [33]. In particular, the late enhancement of EEG delta activity in the VTA and SN may be explained by the stimulation of DA autoreceptors in these areas by residual APO [32]. In contrast, a lack of the clusters produced by acute APO in early theta and late delta EEG activities in the 5xFAD transgenic mice chronically pretreated with saline (see plates B and E in Figure 3 and Figure 4) clearly demonstrates tremendous alterations in the post- and pre-synaptic organization of DA receptors in this neurodegenerative pathology. On the other hand, the evident recovery of the acute APO effects in the theta band observed in the 5xFAD mice chronically pretreated with APO (see plates C and F in Figure 1 and Figure 3) allows the suggestion about APO-dependent repairing/compensatory mechanisms associated in particular with DA receptor sensitization [34]. This supposedly may be amplified by the involvement of so-called “priming” mechanisms activated by the repeated pharmacological stimulation of DA receptors [35]. Other perspectives of AD protection/treatment may be connected with the stimulation of mechanisms of neurogenesis revealed in adults [36,37]. Indeed, DA denervation in the mouse brain has been shown to reduce mesenchymal stem cell proliferation in hippocampal zones of neurogenesis [38]. Thus, the repeated stimulation of DA receptors by APO is expected to potentiate newly proliferated cells in the brain [39]. Eliminating the enhanced delta activity in a baseline EEG from the hippocampus in 5xFAD mice by chronic APO is in line with this suggestion (see Figure 2B). Mechanisms of the late enhancement of delta activity in EEGs from DA-containing areas in WT mice after acute APO injection (see Figure 4A) and a lack of this effect in 5xFAD mice seem to be a new target in further AD studies. Furthermore, sufficiently expressed tendencies of delta suppression declining and beta enhancement rising in EEGs in 5xFAD mice chronically pretreated with saline (see B and E in Figure 3 and Figure 4) deserve to be studied as well. In addition, the results of the experiments on WT mice chronically pretreated with APO may allow clarifying the role of chronic APO itself in combined EEG effects observed in 5xFAD mice.

## 5. Conclusions

This study observed evident disturbances in the frequency spectra of EEGs recorded from different brain areas in 5xFAD mice chronically pretreated with saline or APO before and after acute APO (vs. saline) injection as compared with those in WT littermates. The differentiation in chronic APO pretreatment’s influence on baseline EEGs (see Figure 2) from the forebrain (the cortex and the hippocampus) and the midbrain (VTA and SN) seems to be indicative of differences in DA-dependent mechanisms involved in the observed EEG changes in 5xFAD mice. The acute time-dependent APO (versus saline) effects in the frequency spectra of EEGs from different brain areas in WT mice (both early delta activity suppression and the theta–alpha enhancement followed by late delta enhancement and theta suppression) practically disappeared in 5xFAD mice chronically pretreated with saline and recovered in a transformed form in those exposed to chronic APO treatment (see Figure 3 and Figure 4). Given the expression extent of the effects of acute APO injection that can indirectly characterize DA receptor sensitivity, we conclude that chronic pretreatment with APO can normalize, at least in part, this characteristic of DA receptors in 5xFAD mice. The “clustering” of pharmacological EEG effects and their time-dependent transition between classical frequency bands demonstrates the potential of this approach for the analysis of the cerebral neurotransmission affected by neurodegeneration.

## Data Availability

Data are contained within the current article.

## Appendix A

**Table A1:** Time–frequency clustering of EEG effects of acute APO (vs. saline) in the cortex calculated as a ratio of (APO − Saline)/Saline (in %) in WT and 5xFAD mice chronically pretreated with saline (A and B; n = 8 and 9, respectively) and 5xFAD mice chronically pretreated with APO (C; n = 8). Each time interval is characterized by mean values and SEMs (upper and lower rows, respectively); colored cells are significantly (*p* < 0.05, U-test) elevated (pink) and lowered (blue) levels.

Apomorphine vs. Saline (Cortex) WT Mice Chronically Pretreated with Saline (n = 8)																				
A	Delta 1			Delta 2				Theta						Alpha			Beta 1		Beta 2	
Min/Hz	0.5	0.9	1.4	1.8	2.3	2.9	3.4	4.0	4.7	5.4	6.2	7.0	8.0	9.1	10.3	11.8	13.7	16.0	19.3	25.0
10	−2	−5	−4	4	−19	−28	−24	−21	−10	0	7	12	14	13	12	1	5	3	3	7
SE	0.6	1.3	0.3	0.7	1.2	0.3	0.3	0.5	0.2	0.2	0.0	1.3	0.0	0.8	0.5	1.1	0.9	0.7	0.8	0.1
20	−12	2	−5	0	−23	−26	−18	−16	1	6	12	12	10	0	0	0	2	0	0	14
SE	1.5	0.9	0.8	1.5	0.0	0.5	1.3	0.3	0.0	0.3	0.4	1.1	0.9	1.1	0.3	1.3	0.8	1.6	2.4	0.4
30	3	−5	5	−10	−21	−28	−22	−13	1	10	13	10	9	1	−3	0	0	0	1	15
SE	1.0	0.6	0.4	0.8	0.9	1.0	0.8	0.3	0.0	1.1	1.1	0.2	0.0	0.4	1.0	1.4	0.7	0.7	0.9	1.1
40	3	0	1	0	−17	−21	−18	−5	6	10	13	8	0	−1	−12	−11	−4	2	6	18
SE	0.6	0.3	0.3	0.6	0.2	1.0	1.3	1.1	0.2	0.0	0.5	0.2	1.0	0.6	1.2	1.9	0.7	0.5	0.8	1.4
50	0	−1	0	−2	−2	−11	−9	0	4	10	8	8	0	−4	−12	−7	−5	0	2	19
SE	1.3	0.1	0.3	0.1	0.8	0.6	0.9	0.7	0.5	0.5	0.5	0.2	0.2	1.4	0.9	2.1	0.6	0.9	0.8	1.0
60	−7	7	0	−6	−5	4	0	5	3	4	2	1	−1	0	−9	−4	−3	0	6	−1
SE	1.0	0.9	0.3	0.0	1.0	0.3	0.5	0.7	0.2	0.2	0.2	0.0	0.4	0.8	0.5	1.2	0.5	0.9	0.1	0.9
70	9	−1	0	−5	0	0	0	0	1	1	0	−2	0	0	0	0	−2	3	0	0
SE	2.0	0.2	0.0	0.6	0.5	0.5	0.2	0.2	0.3	0.7	0.2	0.0	0.4	0.6	1.4	1.7	0.8	0.0	0.8	1.3
80	−12	−1	−4	−1	1	5	15	2	0	−5	−1	0	0	−4	3	0	1	1	0	−1
SE	0.8	0.7	0.1	0.3	0.0	0.2	0.5	0.2	0.3	0.0	0.7	0.6	0.6	1.0	0.7	0.6	0.7	0.2	0.7	0.2
90	0	11	1	0	6	7	9	4	−5	−3	−2	−2	0	0	0	0	0	0	−1	−7
SE	1.9	0.3	0.4	0.0	0.2	0.3	0.5	0.5	0.3	0.2	0.3	0.7	1.2	0.2	0.7	0.4	0.2	0.2	0.0	0.5
100	2	1	3	0	7	1	11	0	0	0	−7	−8	−1	−4	0	0	2	4	−1	−3
SE	0.8	0.3	0.6	0.7	0.2	0.7	0.3	0.0	0.3	0.4	0.5	0.8	0.6	0.2	0.7	0.6	0.7	0.4	0.2	0.3
110	0	3	2	0	4	5	10	4	0	−6	−2	−6	−1	−6	−1	1	0	0	1	−1
SE	1.7	0.4	1.2	0.0	0.7	0.5	0.2	0.3	0.2	0.2	0.4	0.2	0.0	0.0	0.2	0.0	0.3	0.8	1.5	0.6
120	3	−11	8	0	6	5	1	0	0	1	−1	−7	−1	−3	−9	−6	−5	8	11	1
SE	0.2	0.4	0.2	0.3	0.3	0.3	0.4	0.6	0.3	0.8	0.5	0.9	0.2	0.6	0.7	0.4	0.7	0.0	0.1	0.3
5xFAD mice chronically pretreated with saline (n = 9)																				
B	Delta1			Delta2				Theta						Alpha			Beta1		Beta2	
Min/Hz	0.5	0.9	1.4	1.8	2.3	2.9	3.4	4.0	4.7	5.4	6.2	7.0	8.0	9.1	10.3	11.8	13.7	16.0	19.3	25.0
10	0	−3	1	0	−14	−11	−6	0	0	7	3	4	0	−2	−6	−13	−8	1	10	16
SE	0.4	0.5	0.4	0.0	0.6	0.5	1.3	0.3	0.1	0.9	0.1	0.5	0.4	0.4	0.8	1.0	0.6	0.1	1.1	1.0
20	−3	3	−1	0	−25	−29	−20	−12	0	8	9	4	0	−4	−3	−15	−1	11	15	32
SE	0.9	0.2	1.0	0.2	1.8	2.3	1.4	1.1	0.2	0.4	0.3	1.2	1.2	1.1	1.4	1.9	0.4	0.4	0.3	0.7
30	1	−1	−1	8	−14	−16	−20	−13	−5	0	1	6	0	−11	−10	−16	0	9	16	36
SE	0.0	0.8	0.5	0.4	0.1	1.8	2.6	0.7	0.1	1.0	1.1	1.2	0.6	1.7	1.2	1.0	0.3	1.5	0.4	1.0
40	0	1	−1	13	−4	−19	−20	−7	0	0	3	2	0	−10	−9	−17	−2	8	13	28
SE	0.7	0.1	0.2	0.8	0.7	2.1	1.7	1.5	0.6	0.5	0.9	0.6	0.9	1.7	1.1	1.9	0.1	0.8	0.7	1.2
50	−1	−7	−4	2	−19	−14	−12	−5	−1	9	10	10	1	−6	−11	−14	−1	4	10	23
SE	0.5	0.8	0.2	0.6	1.9	0.7	1.2	0.5	0.0	0.3	0.6	0.9	1.6	1.4	1.0	1.2	0.3	0.3	0.9	1.1
60	0	14	−1	−1	−1	0	0	6	0	2	2	1	−2	−2	−9	−15	−6	−4	0	11
SE	1.4	0.7	0.8	0.2	0.1	0.3	1.1	1.9	0.4	0.1	0.3	0.3	0.6	0.5	1.3	1.1	0.0	0.0	0.9	2.2
70	−2	8	0	−3	−5	−3	1	0	5	2	2	2	0	−3	−1	−7	−1	−1	0	4
SE	0.5	0.7	0.6	0.6	0.1	0.1	1.9	1.0	0.7	0.0	1.1	0.3	0.3	1.6	0.5	0.9	0.3	0.4	2.1	1.1
80	0	0	0	5	0	−1	−4	0	0	0	0	6	0	1	0	−1	−3	0	0	1
SE	0.1	1.1	0.1	1.2	0.2	0.6	0.2	0.1	0.8	0.9	0.6	0.2	0.2	0.5	0.9	0.3	0.0	0.3	0.0	0.5
90	4	0	6	0	4	1	5	3	9	0	0	0	−3	−4	0	−3	−2	−2	−5	−5
SE	1.7	0.2	1.7	0.3	0.5	0.9	0.2	0.1	0.9	0.3	0.7	0.2	0.2	1.5	0.6	0.7	0.4	0.8	0.7	0.8
100	0	2	1	4	3	0	5	9	3	2	0	0	−2	−3	−1	−1	−5	−4	−3	−3
SE	0.1	1.2	1.2	0.0	1.1	0.2	0.9	0.2	0.4	0.7	0.0	0.3	0.2	0.6	0.0	0.4	0.7	0.5	0.3	0.6
110	−7	0	−2	−10	0	4	1	−2	6	0	4	5	3	5	5	2	−2	0	−2	−8
SE	1.1	0.8	0.7	0.3	0.7	0.9	0.6	0.7	0.9	0.6	0.1	1.3	0.6	0.7	0.8	0.7	0.4	0.4	0.9	0.4
120	−3	0	−1	0	0	1	1	1	0	1	0	2	1	4	3	1	0	−2	0	−10
SE	2.9	2.5	2.4	1.4	2.0	1.8	2.2	2.1	2.4	1.7	1.7	1.9	1.6	2.1	1.9	2.5	2.4	2.1	2.0	2.9
5xFAD mice chronically pretreated with apomorphine (n = 8)																				
C	Delta1			Delta2				Theta						Alpha			Beta1		Beta2	
Min/Hz	0.5	0.9	1.4	1.8	2.3	2.9	3.4	4.0	4.7	5.4	6.2	7.0	8.0	9.1	10.3	11.8	13.7	16.0	19.3	25.0
10	−6	1	−17	2	−1	−18	−22	−20	−15	−8	0	2	8	9	3	0	5	9	10	17
SE	1.0	0.7	1.0	0.8	1.7	2.0	1.7	1.6	1.3	1.1	0.2	0.6	0.6	0.4	0.6	0.5	0.1	0.0	0.9	2.1
20	−5	−1	−1	2	−1	−19	−22	−22	−15	−7	5	10	8	11	1	−3	−1	9	11	13
SE	1.1	0.8	0.7	0.1	0.8	2.1	2.3	1.4	1.9	0.6	0.4	0.4	0.3	0.5	1.0	1.7	1.0	0.8	0.7	1.2
30	−1	−8	−4	2	−4	−26	−24	−20	−13	−2	4	12	8	13	3	1	1	7	7	14
SE	0.0	0.3	0.0	0.1	0.1	0.5	1.1	1.2	0.6	0.9	0.6	0.6	0.2	0.2	0.1	0.4	0.6	0.5	0.7	0.8
40	0	0	0	3	−10	−18	−17	−15	−4	0	8	16	9	7	1	−2	−2	0	0	9
SE	0.6	1.5	0.5	0.5	0.6	1.6	2.1	1.0	1.2	0.6	0.2	0.3	1.0	0.4	0.7	0.4	0.1	0.8	0.2	0.4
50	6	5	0	−6	−15	−14	−2	4	12	0	9	12	0	−3	−4	−10	−2	−8	−8	24
SE	0.2	0.3	0.1	0.1	0.5	1.1	0.9	1.1	0.6	0.0	0.0	0.2	0.3	0.0	0.9	0.1	0.3	0.1	0.4	0.2
60	0	0	0	2	0	0	1	0	2	2	7	3	0	0	−9	−7	−6	−5	1	1
SE	0.4	0.3	0.6	0.6	1.3	0.8	0.3	1.1	0.7	0.1	0.2	0.4	0.4	0.1	0.1	0.3	0.6	0.3	0.3	6.4
70	−1	−13	0	0	3	10	6	5	4	0	0	0	−4	0	−1	0	0	−3	−1	0
SE	0.4	0.2	0.8	0.7	0.6	0.5	0.4	0.3	1.0	0.2	1.5	0.2	0.2	0.1	0.3	0.1	0.2	0.4	0.4	0.6
80	0	−1	0	−1	3	−1	0	1	−8	1	0	−1	0	1	−4	−4	0	1	5	0
SE	0.2	0.7	0.5	0.3	0.5	0.5	0.6	1.3	0.0	0.3	0.1	0.2	0.2	0.2	0.2	0.2	0.0	0.0	0.1	0.2
90	0	0	0	0	0	−2	−10	0	−4	−6	0	−2	−5	−1	0	0	1	4	0	12
SE	0.1	0.0	0.7	0.3	0.4	0.3	0.3	0.3	0.7	0.6	0.7	0.5	0.1	0.6	0.2	0.4	0.3	0.1	0.5	0.0
100	4	−2	−4	−1	0	−2	−12	−10	−7	−7	0	0	−1	0	4	0	6	8	4	8
SE	0.1	0.0	0.7		0.4	0.3	0.3	0.3	0.7	0.6	0.7	0.5	0.1	0.6	0.2	0.4	0.3	0.1	0.5	0.0
110	−10	−1	0	−2	0	0	−9	−10	−8	−2	−1	0	0	7	5	0	6	6	4	0
SE	0.3	0.8	0.8	0.5	0.1	0.4	1.2	1.2	0.9	1.2	0.9	0.5	1.2	0.1	0.3	0.4	0.0	0.1	0.7	1.0
120	0	0	−4	−5	−3	−13	−9	−2	−4	1	3	4	2	6	1	0	0	6	1	0
SE	1.0	1.5	1.5	1.0	1.3	1.3	0.1	0.0	0.1	0.5	0.2	0.6	0.3	0.7	0.9	1.2	2.0	1.5	1.2	1.5

**Table A2:** Time–frequency clustering of EEG effects of acute APO (vs. saline) in the hippocampus calculated as a ratio of (APO − Saline)/Saline (in %) in WT and 5xFAD mice chronically pretreated with saline (A and B; n = 8 and 9, respectively) and 5xFAD mice chronically pretreated with APO (C; n = 8). Each time interval is characterized by mean values and SEMs (upper and lower rows, respectively); colored cells are significantly (*p* < 0.05, U-test) elevated (pink) and lowered (blue) levels.

Apomorphine vs. Saline (Hippocampus) WT Mice Chronically Pretreated with Saline (n = 8)																				
A	Delta 1			Delta 2				Theta						Alpha			Beta 1		Beta 2	
Min/Hz	0.5	0.9	1.4	1.8	2.3	2.9	3.4	4.0	4.7	5.4	6.2	7.0	8.0	9.1	10.3	11.8	13.7	16.0	19.3	25.0
10	9	3	2	−9	−15	−27	−19	−16	−10	0	9	8	10	11	5	4	3	0	1	1
SE	0.4	0.5	0.1	0.6	1.4	1.0	0.3	0.6	0.3	0.5	0.5	0.8	0.0	0.4	0.2	0.2	0.0	1.8	1.1	0.8
20	0	0	0	0	−22	−21	−19	−4	1	3	11	6	0	0	0	−3	0	0	0	17
SE	0.6	2.4	0.6	0.9	0.9	0.6	0.1	0.7	0.2	0.7	0.0	0.2	0.2	0.4	1.1	1.0	0.2	1.8	2.4	1.1
30	−11	−6	3	−1	−21	−28	−18	−11	0	9	10	14	9	1	−2	0	0	0	2	18
SE	0.1	0.3	0.1	0.4	0.3	1.2	0.9	0.5	0.5	1.0	0.9	0.2	0.2	0.2	0.5	1.0	0.5	1.0	2.0	0.3
40	−4	−2	1	−2	−17	−13	−6	−6	0	13	9	7	0	2	−17	−8	−1	0	7	15
SE	0.7	0.1	0.9	0.1	1.0	1.7	1.3	0.2	0.2	0.4	0.2	0.2	0.6	0.6	1.1	1.0	0.0	0.6	0.0	1.0
50	−1	0	−2	−1	1	−11	−7	0	5	7	3	7	0	−3	−14	−10	−3	−1	5	21
SE	0.1	0.4	0.5	0.8	0.7	1.2	1.0	0.3	0.5	0.2	0.4	0.4	0.8	1.0	1.8	1.4	0.2	1.1	1.2	0.5
60	−1	6	−4	−1	0	2	3	1	1	9	3	−1	−1	1	−14	−1	−2	−1	0	0
SE	0.0	1.4	0.2	0.4	0.8	0.2	0.3	0.3	1.0	0.2	0.2	0.0	0.0	0.4	0.0	1.0	0.9	1.6	0.4	0.3
70	−9	−3	−1	0	7	0	1	0	0	0	0	−3	0	0	0	0	0	1	3	−1
SE	0.5	0.4	0.1	0.6	1.2	0.3	0.1	0.8	0.5	0.6	0.2	0.0	0.0	1.0	0.8	1.2	0.2	0.2	0.1	1.1
80	2	−3	−1	0	11	9	6	0	1	0	−1	−2	0	0	0	−1	1	0	−3	−3
SE	0.2	0.8	0.3	0.1	0.5	0.8	0.1	0.2	0.2	0.6	0.7	0.7	0.4	0.7	1.0	0.0	0.4	0.8	1.1	0.3
90	3	1	0	3	9	17	4	0	−7	1	−2	−2	−3	1	−1	1	−3	0	0	−12
SE	0.8	0.5	0.4	1.2	0.0	0.1	0.1	0.5	0.0	0.2	0.0	0.5	0.4	0.2	0.4	0.0	0.8	0.3	0.1	0.5
100	−1	−1	0	3	13	9	2	2	−1	−1	−4	−2	0	0	0	3	0	0	0	−9
SE	0.6	0.9	0.4	0.0	0.1	0.9	0.0	0.2	0.3	0.5	0.2	0.4	0.0	0.2	0.2	0.2	0.3	0.2	0.3	0.9
110	0	3	0	6	3	11	5	0	0	−1	−3	−2	0	−3	1	4	0	−5	0	−4
SE	0.4	3.5	0.3	0.4	0.2	0.1	0.0	0.3	0.2	0.5	0.2	0.2	0.2	0.2	0.0	0.7	0.0	0.0	0.3	0.0
120	12	−1	−1	8	2	11	4	−3	−4	6	1	−5	−2	0	−13	−7	−2	4	8	−8
SE	2.2	1.2	1.0	1.1	1.1	0.4	1.2	0.3	0.3	0.7	0.0	0.9	0.2	0.6	0.4	0.8	1.4	0.7	0.5	0.7
5xFAD mice chronically pretreated with saline (n = 9)																				
B	Delta1			Delta2				Theta						Alpha			Beta1		Beta2	
Min/Hz	0.5	0.9	1.4	1.8	2.3	2.9	3.4	4.0	4.7	5.4	6.2	7.0	8.0	9.1	10.3	11.8	13.7	16.0	19.3	25.0
10	0	−1	0	0	−14	−9	−16	−3	0	1	1	1	−1	−4	−13	−10	0	9	13	24
SE	0.6	1.7	0.3	0.5	1.0	0.1	1.0	0.8	1.0	0.7	0.2	0.5	0.3	0.8	0.6	0.1	0.1	0.2	0.3	0.3
20	1	−12	0	−4	−20	−30	−19	−11	0	3	5	0	−1	−9	−11	−10	2	17	20	38
SE	0.4	0.9	1.4	0.1	2.3	2.6	2.8	0.9	0.3	0.0	0.7	1.3	0.8	1.8	1.6	0.7	0.3	0.3	0.1	0.7
30	1	−6	0	0	−21	−25	−18	−11	−1	1	0	0	−2	−12	−15	−12	2	18	19	46
SE	0.1	1.8	0.7	0.5	1.9	1.4	2.7	1.2	0.7	1.2	0.9	1.3	1.4	1.5	0.9	0.3	0.8	0.5	0.5	0.9
40	0	−1	0	3	−12	−18	−18	−10	−2	0	1	2	−4	−9	−18	−11	0	12	19	36
SE	0.6	0.4	0.7	0.4	0.6	2.2	1.6	1.5	1.3	0.1	1.0	0.4	0.9	0.5	1.9	1.0	1.0	0.4	0.6	0.9
50	0	0	−4	−6	−18	−21	−15	−10	0	5	9	5	0	−8	−12	−13	1	9	16	31
SE	0.9	0.7	0.7	0.6	2.6	2.0	1.6	0.3	0.5	0.6	0.9	0.7	1.0	0.3	0.5	0.0	0.6	0.5	0.5	0.2
60	−1	−1	0	−7	−4	−3	3	4	0	2	0	0	−4	−5	−13	−11	−3	1	8	20
SE	0.8	0.6	0.0	1.5	1.4	0.3	1.1	0.2	0.6	0.3	0.4	0.4	1.2	0.5	1.7	0.6	0.3	0.9	1.6	1.3
70	0	5	0	−3	−6	0	−2	0	0	2	1	0	0	−6	−11	−6	0	1	4	15
SE	0.3	1.0	0.6	0.9	0.3	1.0	0.5	0.3	1.2	0.8	0.1	0.6	0.5	1.3	0.8	0.4	0.7	0.1	1.7	1.8
80	19	0	−5	1	−3	−1	−6	0	−1	9	−1	−1	−3	−7	−2	−2	0	5	2	9
SE	1.7	0.5	0.0	0.5	0.6	0.1	1.0	0.6	0.0	1.4	0.5	0.4	0.8	0.7	0.5	0.6	0.0	0.6	0.4	0.6
90	7	1	4	0	1	0	0	0	0	0	−1	0	−3	−7	−1	−7	0	0	0	4
SE	0.7	0.3	0.7	0.6	1.0	1.1	1.2	0.0	0.6	0.4	0.4	0.0	0.3	0.9	0.9	1.2	0.1	0.7	0.8	1.1
100	0	2	0	4	−7	6	1	10	0	0	−1	0	−7	−4	−4	−8	−1	0	1	5
SE	0.5	0.1	0.2	1.0	0.6	0.7	0.6	0.2	0.2	0.5	0.0	0.8	0.2	0.7	0.0	0.6	0.0	0.4	0.1	1.9
110	−15	7	0	−1	−1	0	0	−1	0	2	1	1	0	0	0	0	0	0	0	0
SE	1.3	0.8	1.0	0.5	0.6	0.6	0.5	0.1	0.2	0.4	0.4	1.6	0.1	0.1	0.5	0.3	0.1	0.9	0.4	1.7
120	−4	17	0	−2	1	−4	3	0	3	2	−2	1	0	−1	0	1	2	0	0	−4
SE	3.0	2.0	2.5	2.5	2.3	2.3	2.3	2.1	1.8	1.6	2.4	2.2	2.3	1.9	2.3	1.9	2.2	2.4	2.0	2.8
5xFAD mice chronically pretreated with apomorphine (n = 8)																				
C	Delta1			Delta2				Theta						Alpha			Beta1		Beta2	
Min/Hz	0.5	0.9	1.4	1.8	2.3	2.9	3.4	4.0	4.7	5.4	6.2	7.0	8.0	9.1	10.3	11.8	13.7	16.0	19.3	25.0
10	−2	−1	−3	4	−2	−20	−19	−17	−14	−6	0	6	0	5	0	0	8	9	11	9
SE	0.7	0.1	0.8	0.7	0.8	1.9	1.6	1.9	1.3	0.6	0.3	0.3	1.4	0.1	0.7	1.0	0.3	0.2	1.3	1.9
20	2	−2	0	3	−3	−22	−22	−19	−14	−4	1	7	2	7	2	0	3	6	11	9
SE	0.2	0.6	0.1	0.1	0.7	1.3	1.5	1.5	1.3	0.6	0.5	0.4	0.4	0.7	0.9	0.9	0.2	0.4	0.6	0.9
30	−3	−2	0	0	−9	−25	−24	−21	−11	−1	3	9	7	8	13	3	2	6	8	8
SE	1.3	0.7	0.3	0.8	0.1	0.8	0.6	0.8	0.0	0.0	0.1	0.0	0.3	0.9	0.1	0.6	0.7	0.6	0.4	0.7
40	−3	0	−1	0	−1	−17	−18	−12	−9	0	6	11	10	9	4	1	0	1	0	4
SE	0.7	0.4	0.4	0.5	1.4	1.4	2.2	1.5	0.3	1.2	0.5	0.2	0.8	0.3	0.3	0.3	0.6	0.5	1.2	0.5
50	−7	0	4	−1	−13	−15	−7	0	6	0	7	8	0	0	0	−7	0	−1	−3	12
SE	0.7	0.1	0.5	0.2	0.4	0.9	0.6	0.6	0.7	0.2	0.1	0.9	0.1	0.3	0.8	0.5	0.2	0.6	0.6	0.6
60	−5	2	−8	0	0	0	3	0	0	0	2	0	0	3	−2	−6	0	0	3	−1
SE	0.0	0.8	0.6	0.1	0.9	0.7	0.0	1.2	1.0	0.7	0.7	0.5	0.1	0.4	0.5	0.2	0.9	0.3	0.3	5.4
70	0	0	5	−5	3	2	10	1	3	−1	−5	2	−5	2	−1	0	0	−1	0	−6
SE	0.2	1.0	0.3	0.2	0.6	0.4	0.4	1.0	0.2	0.2	0.6	0.7	0.1	2.1	0.0	0.1	0.2	1.0	0.5	0.2
80	1	−1	−4	2	0	−1	1	1	0	−1	0	0	−6	0	0	−1	2	4	1	−1
SE	0.5	0.2	0.1	0.0	1.3	0.3	0.6	0.1	0.7	0.2	0.1	0.8	0.2	1.6	0.3	0.7	0.2	1.7	0.3	0.3
90	−1	1	0	−1	1	−2	−1	−3	−3	−4	0	0	−2	−3	1	−2	3	5	0	7
SE	0.6	0.7	0.0	0.3	0.2	0.2	0.4	0.8	0.2	0.1	0.0	0.2	0.2	0.5	0.5	0.5	0.5	0.0	0.3	0.2
100	8	−4	1	0	1	0	−9	−12	−7	−6	−6	−1	−1	1	8	0	6	6	1	4
SE	0.6	0.7	0.0	0.3	0.2	0.2	0.4	0.8	0.2	0.1	0.0	0.2	0.2	0.5	0.5	0.5	0.5	0.0	0.3	0.2
110	−1	3	−2	0	−3	0	−9	−5	−9	−1	0	0	0	9	0	6	7	4	2	−1
SE	0.6	0.2	0.3	0.8	0.1	0.1	0.7	0.8	0.7	0.7	0.8	0.1	0.9	0.3	0.2	0.3	0.5	0.0	0.1	0.0
120	−11	0	−1	−11	−2	−4	−10	−8	−1	−3	2	4	3	7	9	0	5	7	1	0
SE	0.3	1.2	1.8	1.3	1.1	1.3	1.0	0.1	0.0	0.4	0.8	0.9	0.5	1.3	1.4	1.1	1.5	1.3	2.5	1.1

**Table A3:** Time–frequency clustering of EEG effects of acute APO (vs. saline) in the ventral tegmental area (VTA) calculated as a ratio of (APO − Saline)/Saline (in %) in WT and 5xFAD mice chronically pretreated with saline (A and B; n=8 and 9, respectively) and 5xFAD mice chronically pretreated with APO (C; n = 8). Each time interval is characterized by mean values and SEMs (upper and lower rows, respectively); colored cells are significantly (*p* < 0.05, U-test) elevated (pink) and lowered (blue) levels.

Apomorphine vs. Saline (VTA) WT Mice Chronically Pretreated with Saline (n = 8)																				
A	Delta 1			Delta 2				Theta						Alpha			Beta 1		Beta 2	
Min/Hz	0.5	0.9	1.4	1.8	2.3	2.9	3.4	4.0	4.7	5.4	6.2	7.0	8.0	9.1	10.3	11.8	13.7	16.0	19.3	25.0
10	0	−20	−1	−1	−24	−31	−24	−23	−8	0	7	17	15	16	13	11	4	6	0	0
SE	0.1	0.4	0.3	0.4	1.1	3.0	1.6	1.3	0.7	0.1	0.0	0.9	0.7	0.3	0.5	0.8	0.3	0.8	0.7	1.1
20	0	−2	0	−4	−21	−28	−20	−14	0	0	10	19	21	17	9	9	−1	−7	−8	−1
SE	0.5	0.2	0.3	0.6	0.8	1.2	1.4	1.3	1.1	1.3	0.1	0.0	0.4	0.3	0.2	0.0	0.3	1.7	1.0	0.8
30	0	−3	−1	−17	−26	−28	−21	−13	0	10	13	15	11	10	4	7	0	−1	0	10
SE	0.5	0.6	0.5	0.0	1.3	1.6	1.0	0.3	0.5	0.7	0.4	0.3	0.5	0.0	0.9	1.3	0.2	0.8	0.5	0.5
40	9	0	6	−5	−18	−19	−7	−3	3	16	15	5	1	0	−3	−7	−2	−3	0	12
SE	1.1	0.1	0.3	1.5	1.8	1.5	1.0	0.6	0.5	0.7	0.9	0.1	0.3	0.9	0.2	0.8	0.1	0.5	0.4	1.5
50	2	2	−6	−2	−4	−2	−4	1	7	9	10	6	1	−1	−7	−9	−6	−7	0	20
SE	0.0	0.6	0.5	1.1	1.7	1.6	0.2	0.0	0.4	0.6	0.0	0.1	0.1	0.9	0.2	0.3	1.2	1.7	2.2	2.8
60	−1	6	−3	−6	4	1	0	0	0	8	4	5	13	0	−1	−6	−8	−1	−6	−7
SE	0.9	0.5	0.1	0.1	0.5	0.1	0.2	0.1	0.4	0.0	0.1	0.4	1.1	0.3	0.2	0.3	0.3	1.2	0.9	2.9
70	12	0	−5	0	1	0	0	−3	−2	0	−2	3	2	0	1	−1	−1	1	1	0
SE	1.7	0.0	0.9	0.1	0.6	1.5	1.3	1.3	1.1	0.9	0.3	0.7	0.1	0.6	0.9	0.2	0.7	0.7	1.1	2.6
80	2	8	0	−3	7	5	11	−1	4	−5	−2	−3	0	−1	−2	0	−1	0	0	0
SE	0.2	0.6	0.5	0.1	0.6	1.5	2.3	1.3	0.3	1.3	0.1	0.3	0.6	0.3	0.7	0.5	0.3	0.4	0.6	0.5
90	0	0	−3	8	24	19	19	−1	−2	−6	−9	−6	−1	−1	0	0	−1	1	0	0
SE	1.2	1.6	0.1	0.3	1.4	1.0	1.6	0.3	0.3	0.1	0.8	0.0	0.6	0.3	0.0	0.0	0.3	0.5	0.2	0.8
100	0	12	6	2	12	16	14	8	−4	−5	−10	−7	−7	−4	−2	−3	−1	−1	7	2
SE	1.5	1.4	1.5	1.5	0.0	0.5	0.3	0.1	0.1	0.1	0.3	0.9	0.7	0.6	0.7	0.6	0.3	0.2	0.3	0.8
110	3	8	0	−1	14	20	13	2	−6	−8	−8	−9	−8	−1	0	−1	0	0	7	2
SE	2.1	1.9	0.6	0.4	0.2	0.2	0.4	0.6	0.3	0.7	0.6	0.0	0.2	0.8	0.2	0.2	0.2	0.6	0.9	0.2
120	5	0	1	10	13	10	10	4	0	−1	−6	−9	−2	−3	−6	−7	−5	3	7	0
SE	0.2	0.9	0.1	0.1	0.3	0.6	0.4	0.4	0.3	0.9	0.9	1.0	0.8	0.7	0.5	0.8	0.3	0.3	0.6	0.3
5xFAD mice chronically pretreated with saline (n = 9)																				
B	Delta1			Delta2				Theta						Alpha			Beta1		Beta2	
Min/Hz	0.5	0.9	1.4	1.8	2.3	2.9	3.4	4.0	4.7	5.4	6.2	7.0	8.0	9.1	10.3	11.8	13.7	16.0	19.3	25.0
10	1	1	3	0	−21	−13	−5	0	1	3	1	−2	−5	0	−2	0	2	1	3	25
SE	1.3	1.0	1.1	0.5	0.5	0.5	0.7	0.2	0.1	0.0	0.4	1.0	0.5	0.4	0.2	0.2	0.2	0.5	0.1	2.2
20	2	−5	6	−7	−30	−32	−19	−11	1	8	2	−1	0	0	−1	0	8	10	17	46
SE	0.7	0.3	1.2	1.8	2.5	3.0	1.9	0.7	0.4	0.2	0.0	1.5	0.6	0.7	0.0	0.2	1.0	1.2	0.2	1.9
30	7	0	12	0	−16	−29	−16	−2	−2	0	0	−4	−9	−5	−12	−6	8	13	24	61
SE	0.8	1.6	1.6	0.6	1.9	2.4	1.5	0.7	0.9	0.9	1.2	1.0	1.5	0.2	0.7	0.7	1.8	0.9	0.7	1.1
40	14	2	10	6	−17	−20	−16	−9	0	1	−5	−6	−15	−12	−13	−6	6	15	28	60
SE	0.4	0.5	0.8	0.5	1.3	1.9	2.0	0.9	1.0	0.7	1.0	0.7	0.9	0.8	0.2	0.6	1.5	0.5	1.2	0.5
50	−11	0	1	−3	−15	−14	−14	−1	0	4	2	4	−1	−1	−8	−4	0	2	13	37
SE	1.6	0.1	0.4	0.8	2.2	1.8	1.2	0.4	0.7	0.1	1.2	0.3	0.0	0.0	0.4	0.4	1.4	0.2	0.6	1.2
60	0	10	2	−13	−5	1	11	9	1	7	4	0	2	−2	−10	−10	−7	−8	−3	13
SE	0.0	1.0	0.1	0.1	0.0	0.7	0.5	0.5	0.2	0.5	0.1	0.3	0.1	1.1	0.1	0.3	0.0	0.6	0.8	3.1
70	1	0	−5	−1	0	1	2	6	8	5	7	2	2	0	0	−6	−6	−11	−8	0
SE	0.1	0.3	1.0	0.4	0.3	0.9	0.6	0.4	0.8	1.3	1.2	0.3	0.6	0.2	0.1	0.1	0.3	0.1	1.2	2.9
80	0	1	4	3	1	0	0	0	0	1	3	0	0	0	0	0	−4	−5	−3	0
SE	0.5	0.1	0.1	0.4	1.1	0.0	0.1	0.1	0.3	0.9	0.1	0.2	1.0	0.2	0.5	0.5	0.4	0.3	2.2	2.9
90	2	10	−3	4	3	8	14	4	4	3	1	0	−1	0	−5	−8	−9	−9	−8	0
SE	0.2	1.5	1.0	1.4	2.3	0.7	1.0	0.0	0.7	0.5	0.6	1.6	0.2	0.1	0.0	0.5	1.5	0.5	0.6	1.0
100	7	1	6	0	3	17	13	9	2	4	−1	−5	−3	−9	−7	−8	−7	−7	0	1
SE	0.7	0.2	1.0	0.7	0.8	0.8	0.6	1.7	0.6	0.9	0.2	0.4	0.2	0.0	0.1	0.6	0.7	1.0	0.7	1.3
110	0	3	0	8	1	−2	1	−1	4	0	2	0	0	0	0	0	−6	0	−1	0
SE	0.7	0.3	0.3	0.7	0.3	0.7	0.4	0.8	0.8	1.1	0.4	0.1	0.3	0.5	0.9	0.1	0.6	0.3	0.6	1.5
120	3	0	0	5	6	0	7	2	1	0	0	1	1	0	0	−3	−5	−4	−5	−8
SE	2.4	1.8	1.9	1.8	1.9	2.8	2.0	2.5	1.9	1.1	1.4	1.5	1.3	2.4	2.1	2.3	2.1	1.9	1.5	2.1
5xFAD mice chronically pretreated with apomorphine (n = 8)																				
C	Delta1			Delta2				Theta						Alpha			Beta1		Beta2	
Min/Hz	0.5	0.9	1.4	1.8	2.3	2.9	3.4	4.0	4.7	5.4	6.2	7.0	8.0	9.1	10.3	11.8	13.7	16.0	19.3	25.0
10	0	−4	−3	0	−11	−18	−25	−30	−16	−8	−3	0	6	5	5	2	8	16	16	19
SE	0.6	1.8	0.2	0.1	1.6	2.2	2.7	2.0	1.2	1.5	0.9	0.5	0.8	1.0	0.4	0.7	0.2	0.7	0.7	1.3
20	−4	−4	2	0	−13	−27	−29	−27	−16	0	6	12	9	9	2	−2	3	11	13	15
SE	0.5	0.7	0.5	0.2	1.2	1.8	1.8	2.1	1.6	0.7	0.5	0.0	0.8	0.9	0.7	0.2	0.4	0.7	1.0	0.1
30	−1	−5	1	0	−12	−29	−29	−24	−16	−6	6	14	14	16	7	3	8	9	6	9
SE	0.2	0.1	0.2	0.1	0.5	0.9	0.9	1.0	1.0	0.6	0.6	0.7	1.3	0.4	0.3	0.5	0.2	0.9	0.2	1.4
40	−18	−2	1	4	−16	−24	−22	−18	−9	2	13	20	14	14	10	0	0	1	−2	3
SE	1.2	1.0	0.2	0.5	0.9	2.1	2.5	2.1	1.6	0.9	0.5	0.6	0.2	0.7	0.2	0.1	0.6	0.1	1.2	0.2
50	0	0	1	−9	−20	−19	−4	2	15	7	15	16	3	5	−3	−10	−6	−7	−8	9
SE	0.4	0.1	1.0	0.1	1.3	2.3	1.4	1.3	0.6	0.2	0.1	0.3	0.1	0.4	0.5	0.2	0.3	0.3	0.5	0.4
60	−2	3	−1	0	−1	1	5	0	9	12	8	2	2	0	−7	−13	−7	−4	−1	−1
SE	0.6	0.2	0.8	0.7	1.6	0.6	0.7	0.4	0.6	0.5	0.6	0.8	0.9	1.4	0.3	0.3	0.1	0.4	0.7	3.8
70	0	6	6	−1	2	10	9	4	8	3	0	0	−1	0	−3	−6	−8	−2	0	−6
SE	0.7	0.5	0.0	0.0	0.1	0.7	1.1	1.1	1.2	0.9	0.1	0.1	0.5	0.2	0.3	1.2	0.1	0.1	0.5	1.4
80	0	1	13	−3	3	0	0	1	−1	−1	0	−3	0	0	−5	0	0	0	0	1
SE	0.4	0.3	0.1	0.4	0.1	0.6	0.2	0.1	0.0	0.6	0.0	0.2	0.3	0.1	0.2	0.6	0.2	0.2	0.2	0.1
90	0	0	0	6	0	−1	−1	−8	−6	−6	−1	−6	−5	−4	−1	0	4	8	1	14
SE	0.3	0.1	0.0	0.0	0.3	0.2	0.4	0.0	0.2	0.1	0.9	0.8	0.3	0.5	0.8	0.4	0.8	0.4	0.5	0.8
100	0	0	−5	0	−1	−2	−6	−11	−10	−10	−7	−3	−4	0	3	0	12	11	5	12
SE	0.1	0.3	0.2	0.2	0.8	0.5	0.3	0.1	0.4	0.6	0.4	0.5	0.4	0.6	0.3	0.3	0.2	0.9	0.1	0.4
110	1	−4	−9	0	0	0	−4	−2	−7	−2	−2	−2	−1	3	3	0	6	9	2	1
SE	0.4	0.4	0.8	1.0	0.1	0.6	0.5	1.1	1.2	1.1	0.9	0.2	0.9	0.1	0.1	0.2	0.3	0.4	0.1	1.0
120	−2	−6	0	−5	−2	−2	−1	−1	2	0	1	5	7	9	0	1	4	3	−5	−7
SE	0.7	0.1	0.6	1.3	0.8	0.9	0.9	0.7	0.2	0.5	0.0	0.3	1.0	0.7	2.0	0.8	1.2	2.3	0.7	2.3

**Table A4:** Time–frequency clustering of EEG effects of acute APO (vs. saline) in the substantia nigra (SN) calculated as a ratio of (APO − Saline)/Saline (in %) in WT and 5xFAD mice chronically pretreated with saline (A and B; n=8 and 9, respectively) and 5xFAD mice chronically pretreated with APO (C; n = 8). Each time interval is characterized by mean values and SEMs (upper and lower rows, respectively); colored cells are significantly (*p* < 0.05, U-test) elevated (pink) and lowered (blue) levels.

Apomorphine vs. Saline (SN) WT Mice Chronically Pretreated with Saline (n = 8)																				
A	Delta 1			Delta 2				Theta						Alpha			Beta 1		Beta 2	
Min/Hz	0.5	0.9	1.4	1.8	2.3	2.9	3.4	4.0	4.7	5.4	6.2	7.0	8.0	9.1	10.3	11.8	13.7	16.0	19.3	25.0
10	0	−1	0	0	−15	−22	−22	−15	−1	0	8	14	10	15	12	4	3	0	−1	−6
SE	0.4	0.3	0.4	0.3	0.9	0.6	0.6	0.1	0.3	0.3	0.4	1.8	0.7	0.4	0.2	0.7	0.2	0.8	0.3	0.4
20	6	0	2	−1	−14	−23	−14	−10	0	2	8	11	15	14	6	1	0	−3	−4	−2
SE	0.1	0.8	0.3	0.0	0.1	0.6	0.1	0.6	0.3	0.6	0.9	1.5	0.7	0.4	0.5	0.5	0.3	1.1	0.1	0.5
30	−7	0	2	−16	−19	−30	−19	−4	0	1	16	10	10	11	3	3	1	0	0	2
SE	0.0	0.4	0.5	0.5	0.3	0.5	0.0	1.2	0.8	1.2	1.2	0.3	0.1	0.5	0.5	0.2	0.0	0.5	0.4	0.5
40	−12	−4	1	−2	−13	−20	−6	−1	10	8	12	1	2	0	−5	−5	0	−3	0	14
SE	1.0	0.0	0.4	0.0	1.0	0.4	0.3	0.6	1.1	0.9	0.6	1.0	0.6	0.7	0.2	0.2	0.5	0.7	0.4	0.1
50	13	−8	0	−1	0	−3	−3	1	0	11	2	2	0	−7	−5	−7	−1	−1	0	13
SE	0.3	0.8	0.6	0.1	0.9	0.0	1.0	1.1	1.8	0.5	1.6	0.1	0.9	0.9	0.3	0.2	0.2	0.2	0.9	0.4
60	1	0	−13	0	5	0	1	0	−2	3	2	0	0	0	−2	−6	−1	0	0	6
SE	0.6	0.3	0.1	0.1	0.6	0.4	0.1	0.6	0.8	0.9	0.0	0.5	0.3	0.0	0.0	0.5	0.6	0.5	0.7	0.2
70	−3	0	−4	4	10	5	1	−1	−3	−4	0	0	0	0	−1	−1	0	0	0	3
SE	0.9	1.0	0.6	0.1	0.5	0.3	0.0	0.8	0.1	0.2	0.4	0.7	0.9	1.2	0.8	0.9	0.9	0.8	0.3	1.0
80	−1	1	−1	0	1	17	19	1	0	−1	−6	−8	−1	−4	−1	−1	−1	1	0	4
SE	0.2	0.9	0.3	0.8	0.3	0.1	1.0	0.2	0.4	0.6	0.6	1.2	0.6	0.3	0.6	0.2	0.0	0.2	0.0	0.0
90	−1	−1	0	12	20	11	15	2	−2	−4	−7	−6	−1	−8	−4	−2	0	0	3	6
SE	0.5	2.4	0.1	1.0	0.6	0.0	0.3	0.6	1.4	0.0	0.0	0.1	0.3	0.0	0.9	0.5	0.0	0.2	0.3	0.7
100	0	−1	3	5	14	15	18	8	−3	−6	−10	−9	−12	−9	−1	−1	0	1	7	7
SE	0.5	1.0	0.6	0.3	0.3	1.5	0.9	0.3	0.3	0.3	0.9	0.4	0.6	0.3	0.3	0.5	0.5	0.3	0.6	1.0
110	9	0	0	4	15	17	14	1	−9	−6	−10	−13	−4	−5	−7	0	0	1	7	6
SE	2.1	0.5	0.4	0.3	0.9	0.7	1.5	0.2	0.4	0.2	0.0	0.3	0.1	0.3	0.3	0.2	0.7	0.2	0.4	0.5
120	3	5	0	1	12	21	17	8	0	−3	−5	−6	−6	−8	−5	−7	−5	1	2	1
SE	0.0	0.6	0.5	0.1	0.1	0.4	0.1	0.1	0.4	0.6	0.3	0.4	1.0	0.6	0.5	0.3	0.5	0.8	0.0	0.5
5xFAD mice chronically pretreated with saline (n = 9)																				
B	Delta1			Delta2				Theta						Alpha			Beta1		Beta2	
Min/Hz	0.5	0.9	1.4	1.8	2.3	2.9	3.4	4.0	4.7	5.4	6.2	7.0	8.0	9.1	10.3	11.8	13.7	16.0	19.3	25.0
10	3	−2	17	−6	−8	−5	−7	0	1	3	1	0	−8	−8	−12	−7	0	2	9	26
SE	0.6	1.0	2.2	0.1	0.7	0.6	0.7	0.1	0.3	0.1	0.4	0.1	1.4	0.4	0.8	0.3	0.1	0.1	1.4	1.0
20	0	−3	1	1	−15	−24	−19	−2	3	4	2	0	−7	−12	−16	−9	3	10	16	43
SE	0.2	0.9	0.1	1.4	1.4	1.5	2.3	0.0	0.1	0.1	0.6	0.6	0.8	0.8	1.5	0.7	0.4	1.0	1.4	0.1
30	0	0	4	8	−13	−21	−16	−9	3	3	0	−7	−14	−17	−15	−12	5	14	23	50
SE	0.2	0.1	1.5	1.2	1.6	1.7	2.0	0.5	0.5	0.5	0.1	1.6	1.6	1.5	0.8	0.8	1.1	0.7	1.1	0.3
40	4	0	7	13	−6	−11	−9	−5	−1	1	0	−7	−16	−16	−16	−9	2	10	19	44
SE	0.9	0.8	0.5	0.7	0.8	0.8	0.6	1.7	0.6	0.8	1.0	0.7	1.4	0.6	1.3	1.0	0.3	0.9	0.7	0.2
50	−1	−1	2	6	−4	−12	−1	−1	0	5	3	−2	−9	−10	−11	−12	0	5	8	35
SE	0.2	0.6	0.0	0.0	0.8	0.5	0.1	0.8	0.3	0.3	0.6	1.0	0.9	0.1	1.2	0.7	0.6	0.8	0.5	0.7
60	−1	4	15	0	0	0	10	7	9	8	0	0	−7	−8	−12	−13	−5	−3	−2	12
SE	0.2	1.0	1.4	0.5	0.1	0.4	1.3	0.1	0.4	0.4	0.5	0.1	0.5	0.5	0.2	0.4	1.2	0.4	0.4	2.5
70	−1	1	0	−5	1	9	11	9	12	0	4	−6	−2	−3	−3	−11	−5	−3	−4	4
SE	0.5	0.5	0.2	0.4	1.9	0.4	0.2	0.6	0.7	0.4	0.9	0.3	0.0	0.4	0.8	0.7	0.4	0.2	0.3	1.2
80	−1	10	1	8	0	0	0	2	2	1	0	1	−5	−1	−1	−1	−2	−1	−3	0
SE	0.7	0.6	1.4	0.1	0.3	0.0	0.1	0.5	0.1	0.1	0.5	1.0	0.0	0.8	0.1	0.6	1.4	0.7	0.3	1.2
90	0	8	3	−2	2	3	14	11	8	1	0	−2	−3	−4	−4	−9	−8	−6	−4	4
SE	0.0	0.2	0.9	0.1	1.2	0.6	1.4	1.2	1.2	0.0	0.3	1.2	0.1	0.1	0.1	0.8	0.5	1.0	0.5	1.0
100	1	5	0	−1	0	5	10	7	4	5	−1	−8	−1	−2	−7	−1	−2	−1	−3	0
SE	0.5	0.0	0.0	0.4	0.1	0.6	0.7	0.5	0.4	0.9	0.4	0.1	0.1	0.2	0.4	1.0	0.1	0.1	0.4	0.2
110	6	2	1	0	0	−1	0	3	0	2	0	2	0	0	−1	−1	−1	−1	−3	−1
SE	0.7	0.4	1.5	0.1	0.3	0.9	0.6	0.0	0.1	0.1	0.7	0.7	0.3	0.1	0.6	0.9	0.8	0.4	0.4	0.4
120	−1	0	4	−1	4	0	7	0	1	0	0	2	−1	0	−1	−1	−1	0	−2	−1
SE	2.0	2.2	2.1	2.0	2.2	2.2	1.8	1.9	1.4	1.7	1.6	1.7	1.9	1.7	2.5	2.4	2.0	1.8	1.5	1.6
5xFAD mice chronically pretreated with apomorphine (n = 8)																				
C	Delta1			Delta2				Theta						Alpha			Beta1		Beta2	
Min/Hz	0.5	0.9	1.4	1.8	2.3	2.9	3.4	4.0	4.7	5.4	6.2	7.0	8.0	9.1	10.3	11.8	13.7	16.0	19.3	25.0
10	−3	0	−3	1	0	−18	−17	−20	−15	−11	0	0	2	0	0	0	5	13	16	13
SE	0.2	0.7	0.7	0.0	0.3	2.0	1.4	1.7	0.5	0.7	0.9	0.4	0.9	1.1	0.9	1.3	0.4	1.0	0.7	2.5
20	−2	−1	−1	0	0	−19	−19	−21	−14	−3	2	5	1	0	0	−1	0	10	15	11
SE	0.3	0.4	0.3	0.0	1.0	1.5	1.7	1.9	1.6	0.4	0.4	1.3	1.6	0.7	1.5	1.0	0.8	0.3	0.8	0.1
30	0	−9	−2	2	−9	−15	−22	−22	−12	−9	1	10	11	6	6	0	1	9	8	10
SE	0.3	0.2	0.1	0.5	0.1	0.0	0.4	1.1	0.6	0.5	0.1	0.4	0.1	0.2	0.1	0.1	0.4	0.5	0.4	0.4
40	−4	−2	−1	6	−3	−15	−19	−16	0	0	9	13	8	9	1	−4	−5	1	0	9
SE	1.3	1.4	0.3	0.3	1.0	0.7	1.7	1.7	0.9	1.3	0.6	0.3	0.7	0.9	0.9	0.4	0.2	0.4	0.2	0.7
50	−8	−2	0	−10	−6	−14	−1	0	13	3	10	14	0	−3	−5	−13	−5	−8	−5	21
SE	0.5	1.2	0.0	0.1	0.0	1.1	1.2	1.4	0.3	0.5	0.0	0.2	0.9	0.7	0.4	0.3	0.5	0.7	0.7	0.9
60	10	1	0	0	0	−2	0	4	3	1	4	3	1	0	−10	−7	−7	−5	0	2
SE	0.3	0.1	0.8	0.7	0.0	0.1	0.4	0.2	0.8	1.2	0.7	1.3	0.7	0.8	0.6	0.0	0.2	0.2	0.5	6.3
70	−1	2	3	0	6	1	1	1	8	8	1	1	0	0	−7	−2	−4	−5	−1	−5
SE	1.0	0.5	0.1	0.8	0.4	0.4	1.2	0.5	0.3	0.8	0.6	0.7	0.4	0.7	0.4	0.3	0.6	0.4	1.4	1.7
80	−1	0	1	2	−4	0	−1	−2	−2	−3	0	1	0	−1	0	−2	0	3	2	1
SE	0.3	0.5	0.7	0.2	0.2	0.4	0.0	0.3	1.0	0.4	0.4	0.4	0.1	1.3	0.6	1.3	0.2	0.0	0.4	0.4
90	6	−6	−1	2	−1	−1	−1	−1	−2	−4	−2	1	−6	−3	−8	−3	0	4	2	14
SE	0.2	0.1	0.4	0.1	0.4	0.0	1.2	0.8	0.5	0.3	0.5	0.7	0.1	0.9	0.0	0.4	0.1	0.7	1.5	0.1
100	−6	−1	0	0	0	−5	−8	−13	−9	−6	−6	−2	−2	2	0	0	7	7	6	11
SE	0.4	0.4	0.6	0.1	0.4	0.0	0.0	0.1	0.7	0.0	0.4	0.3	0.2	0.3	0.3	0.2	0.4	0.1	0.2	1.5
110	3	−3	2	0	1	1	−3	−8	−8	−7	−3	−4	−1	1	1	5	1	6	5	0
SE	1.2	0.2	0.5	0.6	0.2	0.7	0.4	1.2	1.2	0.8	0.4	0.8	0.9	0.0	0.5	0.4	0.0	0.3	0.3	1.3
120	−3	0	0	−1	0	−4	−2	0	0	0	0	6	3	6	3	0	0	2	0	−3
SE	2.4	0.6	1.4	0.5	0.9	1.7	0.1	0.6	0.0	0.9	0.8	0.3	1.0	1.5	0.9	1.8	2.0	1.8	2.2	3.4
